# Effects of Integrated Care on Disease-Related Hospitalisation and Healthcare Costs in Patients with Diabetes, Cardiovascular Diseases and Respiratory Illnesses: A Propensity-Matched Cohort Study in Switzerland

**DOI:** 10.5334/ijic.2455

**Published:** 2016-04-08

**Authors:** Carola A. Huber, Oliver Reich, Mathias Früh, Thomas Rosemann

**Affiliations:** Department of Health Sciences, Helsana Group, Zürich, Switzerland; Institute of General Practice, University of Zürich, Zürich, Switzerland

**Keywords:** integrated care, hospitalization, costs, chronic diseases

## Abstract

**Background:**

There is an ongoing discussion on the further promotion of integrated care models in many healthcare systems. Only a few data, which examine the effect of integrated care models on medical expenditures and quality of care in chronically ill patients, exist.

**Aims:**

To investigate the effect of integrated care models on disease-related hospitalisations as a quality indicator and healthcare costs in patients with either diabetes, cardiovascular diseases or respiratory illnesses.

**Methods:**

A propensity-matched retrospective cohort study based on a large Swiss health insurance database (2012–2013) was performed for three chronic patient groups (diabetes, cardiovascular diseases, respiratory illnesses), who were enrolled in an integrated care model and compared to individuals in a standard care model. Multivariate regression models were applied to estimate the effect of integrated care models on disease-related hospitalisations and healthcare costs.

**Results:**

The matched cohorts included a total of 12,526 patients with diabetes, 71,778 with cardiovascular diseases and 17,498 with respiratory illnesses, in which each one half was enrolled in integrated care models and the other half in standard care models. Diabetes and cardiovascular patients with integrated care models had a significantly lower probability of disease-related hospitalisation compared to those with standard care models (*p* < 0.01). Healthcare costs were statistically significant lower in all three patient groups with integrated care, but with the highest effect in patients with diabetes (Swiss francs (CHF) –778).

**Conclusions:**

Integrated care may provide an effective strategy to improve the quality of care and to reduce healthcare costs in chronically ill patients. Study findings intend to contribute to the ongoing political discussion on integrated care and provide evidence for improved and more effective care of patients with chronic diseases.

## Introduction

Switzerland has almost 20 years of experience in the development of integrated care models [1] and provides a wide range of insurance plans. In 2013 nearly 60% of the Swiss population were enrolled in integrated care models [2]. But despite this high percentage of enrolees, there is an ongoing discussion on the further promotion of integrated care models in the Swiss healthcare system. The reason for this discussion is the existence of different types of integrated care models. Contracted capitated models suggest the greatest potential for efficient care coordination, but only about a quarter of all insured persons are enrolled in this type of health plan [3]. Overall, integrated care models are seen as possible interventions to improve the quality in healthcare use and to reduce medical expenditures, especially in patients with chronic diseases. However, there is little international as well as national evidence on the improved healthcare quality and the cost-effectiveness of managed care models among chronically ill patients. A recent published meta-review suggests a beneficial effect of integrated care programmes on some outcomes, including hospital admissions and quality of care, in adults affected by chronic conditions [4]. A few reviews reported reduced costs [4]. Since the existing studies were often performed in fully integrated healthcare settings (e.g. US health maintenance organisations), the results are not directly transferable to other healthcare settings. In Switzerland, only a few studies investigated the effect of integrated care on medical expenditures and healthcare use. These suggested an economic efficiency of selected integrated care models in different Swiss healthcare settings, exist [5,6,7,8,9,10]. Furthermore, little is known about the effect of integrated care on the quality of care, especially in patients with chronic diseases [10,11]. The implementation of integrated care exerts great potential for coordination advantages and is, thus, particularly useful for patient groups suffering from highly prevalent chronic diseases. There is strong evidence that diabetes mellitus, cardiovascular diseases and respiratory illnesses are one of the most prevalent and costly chronic diseases worldwide [12,13,14]. Chronically ill patients have a high probability to be hospitalised and to incur high medical costs. The management of these patient groups can be seen as an effective strategy to avoid hospitalisations and to reduce healthcare costs.

This study aimed to assess the impact of integrated care models on hospital admissions and to determine potential cost reductions from integrated care in patients affected with highly prevalent chronic diseases including diabetes mellitus, cardiovascular diseases and respiratory illnesses. Using a propensity-matched retrospective cohort of patients with diabetes, cardiovascular diseases and respiratory illnesses, we compared the probability of disease-related hospitalisation, reflecting quality of care, and the incurred healthcare costs after 1 year between patients who were and were not enrolled in integrated care models.

## Methods

### Study design and population

We performed a cohort study embedded in insurance claims data from the Helsana health insurance group. Helsana is the leading health insurer in Switzerland covering about 1.2 million mandatory insured persons. The patient-level linked database included longitudinal information on sociodemographics, health insurance status, prescribed drugs, healthcare utilisation and its associated costs. Data also included information on health insurance models. Insured persons can choose between a standard care model, which allows the freedom of choice and unlimited access to physicians in the outpatient setting, and an integrated care model, but receive in return of it the benefit of premium reduction. In this study we used data of patients continuously enrolled between 2012 and 2013 in contracted integrated care model with capitation. In contracted models with capitation, a network of physicians corporates with the health insurer [6]. These networks comprise either a group of independent individual (general) practitioners or a Health Maintenance Organisation. The network assumes collective responsibility for a financial budget which consists of per capita funding calculated by the health insurer. Within this remuneration by capitation scheme, the network members provide healthcare for the contractually insured persons for 1 year. However, this budget is rather virtual than paid per se since the providers are paid by the nationwide fee-for-service scheme and do not receive a defined periodic payment. In case the virtual budget falls below the negotiated limit after the year, the remaining difference will be split between the network and the health insurer. Otherwise, if the budget is exceeded, the negative difference will be also divided. Besides the mentioned economic responsibility, the capitation model has in the first instance a high medical responsibility for the whole treatment process, primarily regarding the interface management between the different specialists and gatekeepers in the outpatient setting as well as between the different (inpatient) institutions.

Overall, there are two important aspects regarding coordinated and integrated care. The first one is the gatekeeping aspect, which refers to the coordination at the beginning of the treatment process. Patients enrolled in an integrated care model have to initially refer to their chosen general practitioner, when they seek medical aid. The second one is the coordination aspect during the treatment process, which appears after the entry into the healthcare system by further referrals to specialists or hospitals.

Our cohort includes men and women aged 18 years or older who were identified as patients with diabetes, cardiovascular diseases and respiratory illnesses in 2012. Patients with chronic diseases were identified by an inpatient International Classification of Diseases, Version 10, German Modification, diagnosis or by the ‘WHO Anatomical Therapeutic Chemical code’ of prescribed drugs (outpatient setting). A patient was, therefore, identified as having diabetes when the person either was diagnosed with diabetes by a hospital discharge record according to the International Classification of Diseases, Version 10, classification system (E10–E14) or had been prescribed any anti-diabetic drug (WHO Anatomical Therapeutic Chemical code: A10) in the outpatient setting in 2012. Using prescribed drug data as proxies for diagnoses from the outpatient setting is considered a valid approach in the literature [15,16,17,18]. Our classification system for the identification of patients with diabetes as well as with cardiovascular diseases and respiratory illness is described in Appendix Table A1. Since patients enrolled in an integrated care model may differ in baseline characteristics from patients in a standard care model, we performed propensity score matching for each patient group with chronic disease to balance observed covariates and to adjust for confounding. Propensity score matching is increasingly being used to reduce the impact of selection bias when estimating causal treatment effects using observational data [19]. To calculate the propensity score for each patient group, we fitted a logistic regression model in which the outcome was the probability of enrolment in an integrated care model, predicted by patient’s age, sex, region of residence, type of cost-sharing (deductibles: low (Swiss francs (CHF) 300/500) versus high (Swiss francs (CHF) 1000–2500), number of hospital days in the year before and comorbidity. Patients’ comorbidity was measured by an updated Chronic Disease Score. The Chronic Disease Score is a proxy measure of severity of chronic morbidity, based on dispending pharmacy data validated in a Swiss population and is divided into five levels (‘low’ (level 1); ‘middle’ (levels 2 and 3); ‘high’ (levels 4 and 5)) [20]. By using patients’ individual propensity score, patients enrolled in an integrated care model were matched to an equal number of those with a standard care model, who had the closest estimates of propensity score (nearest neighbour method, 1:1 matching). The adequacy of the matching was checked by two approaches: visual inspection and calculating efficiency measures [21,22]. For visual inspection, we displayed the distribution of propensity score and performed histograms of propensity scores before and after matching. For the numerical inspection, we calculated measures which reflect the effectiveness of the propensity score matching: (1) summary of balance for all (original) data, (2) summary of balance for matched data and (3) percent balance improvement. The first and second steps provide the mean differences in the density of the covariates for the treatment (integrated care) and control group (standard care model) before and after matching (percent balance). The last step gives the mean difference by comparing the mean difference for all data with the mean difference for matched data (percent balance improvement).

### Outcomes

Disease-related hospitalisations in the follow-up year (2013) were defined for all three patient groups as having at least one given disease-related diagnosis at hospital discharge in 2013 according to the International Classification of Diseases, Version 10, classification system. For patients identified with diabetes in 2012, each hospitalisation associated with diabetes in 2013 was taken into account. Furthermore, common complications in patients with diabetes related to ischaemic heart diseases, cerebrovascular diseases, renal failure, glomerular disorders in diabetes, diabetic cataract, diabetic retinopathy, atherosclerosis, diabetic peripheral angiopathy, diabetic mononeuropathy and diabetic polyneuropathy were included. Hospitalisations related to cardiovascular diseases were defined as hospitalisations caused by hypertensive diseases, ischaemic heart diseases, pulmonary heart disease and diseases of pulmonary circulation, paroxysmal tachycardia, atrial fibrillation and flutter, other cardiac arrhythmias, heart failure, cerebrovascular diseases and diseases of arteries, arterioles and capillaries. Following complications in patients with respiratory illnesses were considered: simple and mucopurulent chronic bronchitis, unspecified chronic bronchitis, emphysema, other chronic obstructive pulmonary disease, asthma, status asthmaticus and bronchiectasis. If one of these complications in each patient group (diabetes, cardiovascular diseases, respiratory illnesses) was recorded as the primary or secondary diagnosis in the inpatient claims, the hospitalisation was specified as disease-related. The classification for the three chronic groups by the International Classification of Diseases, Version 10, codes is shown in Appendix Table A2.

Total healthcare costs were defined as the sum of outpatient and inpatient costs per patient/year (2013). Outpatient costs included payments for office-based physician visits, hospital outpatient visits, paramedical visits, prescription drugs, laboratory tests and medical devices. Inpatient costs included payments for hospitalisations, rehabilitation, nursing and emergency transport services. Costs associated with hospitalisation such as drugs or medical drugs were routinely included in the charge for these hospitalisations.

### Statistical analysis

Based on the propensity score-matched sample, we analysed the matched pairs using frequency tables. Descriptive results are given as number (percentage) of patients for categorical variables and as mean values with standard deviation for numeric variables. Logistic regression models were applied to estimate the effect of integrated care on disease-related hospitalisations for all three patient groups. Multiple linear regression analysis was performed to determine the impact of integrated care on healthcare costs among chronically ill patients. Given the skewed nature of the cost distribution, we used generalised linear models with negative binomial distribution and with linear link function, which provided absolute values in Swiss Francs (CHF) as estimates. We adjusted the models for age, sex, region of residence, deductibles, comorbidity and the number of hospitalised days in the year before. All analyses were performed using the statistical program ‘R’.

## Results

**Figure A1:**
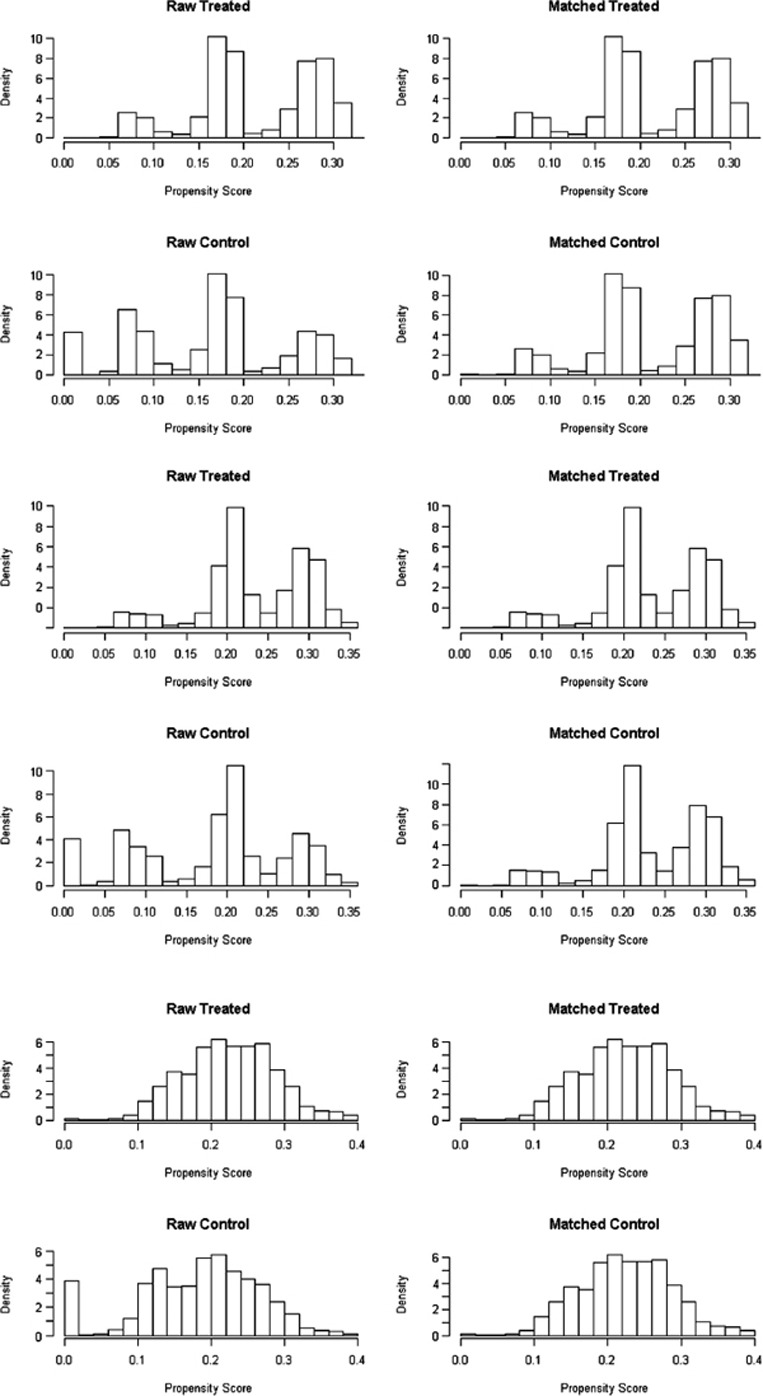
Propensity score before and after matching in patients with diabetes, cardiovascular diseases and respiratory illnesses.

**Figure A2:**
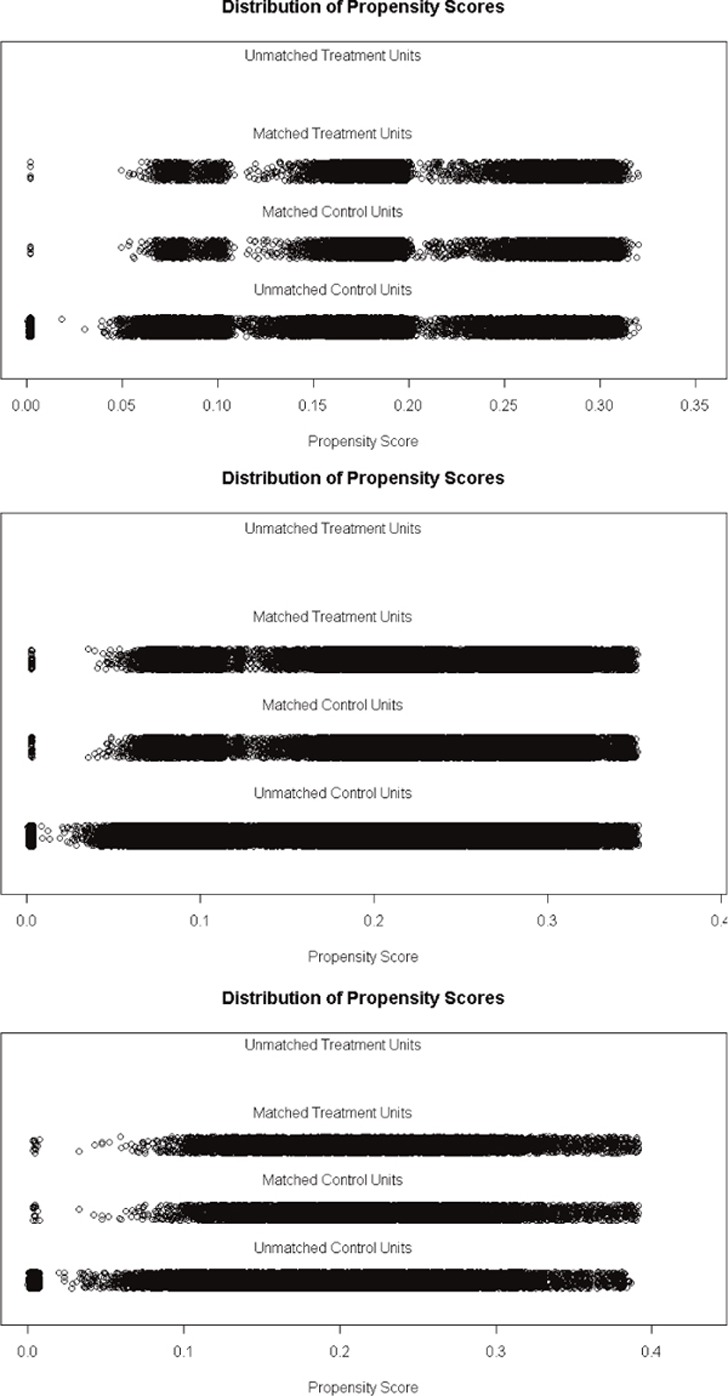
Distribution of propensity score in patients with diabetes, cardiovascular diseases and respiratory illnesses.

We identified a total of 36,532 patients with diabetes, 186,986 with cardiovascular diseases and 45,364 with respiratory illnesses from source population at baseline. Population characteristics before matching are shown in Appendix Table A3. After applying propensity matching, we included 12,526 patients with diabetes, 71,778 with cardiovascular diseases and 17,498 with respiratory illnesses. Results from calculating covariate balancing before and after matching showed that the adequacy of the matching was given (Appendix Tables A4, A5, A6; Figures A1–A2). In the matched samples, differences in the distribution of the covariates between the patient group with integrated care models (treated group) and those with standard care models (control group) were much smaller for most of the variables. The matching considerably improved their balance. Population characteristics from the propensity-matched sample for all three patient groups are shown in Table 1. In each patient group, the number of persons with an integrated care model was equal to the number of persons with a standard care model. The patient group with diabetes consisted of a higher proportion of men (56%), included about 58% of patients aged between 65 and 84 years, and the majority had a high level of comorbidity (levels 4 and 5: 57%). Among patients with cardiovascular diseases, the proportion of men was slightly lower (47%), the mean age was 68 years and more than half had a comorbidity level of 3 or 4 (54%). Patients with respiratory illnesses included about 42% of patients aged younger than 55 years, more than half were women (56%) and most had middle to high comorbidity level (50%).

**Table 1:** Patients’ characteristics by chronic disease after propensity matching.

Characteristic	Patients with diabetes *n* = 12,526	Patients with cardiovascular diseases *n* = 71,778	Patients with respiratory illnesses *n* = 17,498
	*n* (%)	*n* (%)	*n* (%)

Population characteristics (2012)			
Male	7043 (56.2)	33,716 (47.0)	7686 (43.9)
Mean age (sd)	67.1 (12.7)	67.9 (13.5)	56.7 (18.6)
Age group (years)			
18–44	677 (5.4)	4063 (5.7)	4848 (27.7)
45–54	1236 (9.9)	7307 (10.2)	2551 (14.6)
55–64	2683 (21.4)	13,894 (19.4)	2988 (17.1)
65–74	4082 (32.6)	21,536 (30.0)	3797 (21.7)
75–84	3167 (25.3)	18,823 (26.2)	2629 (15.0)
≥85	681 (5.4)	6155 (8.6)	685 (3.9)
Region of residence			
Lake Geneva	823 (6.6)	3666 (5.1)	1986 (11.4)
Mittelland	2586 (20.7)	14,745 (20.5)	3768 (21.5)
Northwest	3240 (25.9)	17,571 (24.5)	3919 (22.4)
East	2628 (21.0)	14,982 (20.9)	2808 (16.1)
Ticino	10 (0.1)	66 (0.1)	26 (0.2)
Central	465 (3.7)	2727 (3.8)	691 (4.0)
Zurich	2774 (22.2)	18,021 (25.1)	4300 (24.6)
Health insurance status			
Care model			
Standard care model	6263 (50.0)	6263 (50.0)	6263 (50.0)
Integrated care model	6263 (50.0)	6263 (50.0)	6263 (50.0)
Deductible			
High (>CHF500)	608 (4.9)	7575 (10.6)	2521 (14.4)
Low (CHF300/500)	11,918 (95.2)	64,203 (89.5)	14,977 (85.6)
Chronic Disease Score			
Mean chronic disease score (sd)	7053 (5679.7)	5048 (4808.9)	5702 (5366.3)
Level 1 (0–999)	623 (5.0)	11,757 (16.4)	3437 (19.6)
Level 2 (1000–2499)	2710 (21.6)	12,977 (18.1)	2707 (15.5)
Level 3 (2500–4999)	2102 (16.8)	17,126 (23.9)	3539 (20.2)
Level 4 (5000–9999)	4234 (33.8)	21,347 (29.7)	5169 (29.4)
Level 5 (≥10,000)	2857 (22.8)	8570 (11.4)	2646 (15.1)
Hospitalisation (2011)	2349 (18.8)	12,049 (16.8)	2749 (15.7)
No. of hospitalised days (2011)			
Mean days (sd)	3.2 (12.2)	2.4 (9.7)	2.2 (9.4)
0–3 days	10,604 (84.7)	62,483 (87.1)	15,440 (88.2)
4–10 days	969 (7.7)	4826 (6.7)	1149 (6.6)
>10 days	953 (7.6)	4469 (6.2)	909 (5.2)

sd, standard deviation.

The proportions in population characteristics between persons with integrated care models and those with standard care models were approximately balanced after matching (Table 2).

**Table 2:** Patients’ characteristics by chronic disease and care model (integrated vs standard) after propensity matching.

Characteristic	Diabetes		Cardiovascular diseases		Respiratory illnesses	

	With ICM *n* = 6263	With SCM *n* = 6263	With ICM *n* = 35,889	With SCM *n* = 35,889	With ICM *n* = 8749	With SCM *n* = 8749
	*n* (%)	*n* (%)	*n* (%)	*n* (%)	*n* (%)	*n* (%)

Population characteristics (2012)						
Male	3507 (56.0)	3536 (56.5)	16,913 (47.1)	16,803 (46.8)	3770 (43.1)	3916 (44.8)
Mean age (sd)	67.1 (12.8)	67.1 (12.6)	68.0 (13.5)	67.8 (13.5)	57.1 (18.9)	56.3 (18.3)
Age group (years)						
18–44	355 (5.7)	322 (5.1)	2071 (5.8)	1992 (5.6)	2434 (27.8)	2414 (27.6)
45–54	615 (9.8)	621 (9.9)	3508 (9.8)	3799 (10.6)	1186 (13.6)	1365 (15.6)
55–64	1264 (20.2)	1419 (22.7)	6701 (18.7)	7193 (20.0)	1398 (6.0)	1590 (18.2)
65–74	2073 (33.1)	2009 (32.1)	11,024 (30.7)	10,512 (29.3)	1986 (22.7)	1811 (20.7)
75–84	1634 (26.0)	1536 (24.5)	9594 (26.7)	9229 (25.7)	1390 (15.9)	1239 (14.2)
≥85	325 (5.2)	356 (5.7)	2991 (8.3)	3164 (8.8)	355 (4.1)	330 (3.8)
Region of residence						
Lake Geneva	407 (6.5)	413 (6.6)	1815 (5.1)	1866 (5.2)	952 (10.9)	1032 (11.8)
Mittelland	1297 (20.7)	1290 (20.6)	7340 (20.5)	7393 (20.6)	1877 (21.5)	189 (21.6)
Northwest	1636 (26.1)	1603 (25.6)	8880 (24.7)	8685 (24.2)	1992 (22.8)	1925 (22.0)
East	1307 (20.9)	1321 (21.1)	7506 (20.9)	7465 (20.8)	1442 (16.5)	1365 (15.6)
Ticino	5 (0.1)	6 (0.1)	33 (0.1)	36 (0.1)	13 (0.2)	17 (0.2)
Central	235 (3.8)	232 (3.7)	1370 (3.8)	1364 (3.8)	354 (4.1)	341 (3.9)
Zurich	1376 (22.0)	1397 (22.3)	8945 (27.9)	9080 (25.3)	2119 (24.2)	2179 (24.9)
Health insurance status						
Deductible						
High (>CHF500)	311 (5.0)	297 (4.7)	3844 (10.7)	3731 (10.4)	1276 (14.6)	1245 (14.2)
Low (CHF300/500)	5952 (95.0)	5966 (95.3)	32,045 89.3)	32,158 (89.6)	7473 (85.4)	7504 (85.8)
Chronic Disease Score						
Mean chronic disease score (sd)	7141 (5860.7)	6966 (5491.9)	5122 (4928.4)	4975 (4685.2)	5844 (5560.4)	5561 (5161.5)
Level 1 (0–999)	302 (4.8)	321 (5.1)	5873 (16.4)	5884 (16.4)	1660 (19.0)	1777 (20.3)
Level 2 (1000–2499)	1326 (21.2)	1384 (22.1)	6396 (17.8)	6585 (18.4)	1330 (15.2)	1377 (15.7)
Level 3 (2500–4999)	1061 (16.9)	1041 (16.6)	8544 (23.8)	8581 (23.9)	1782 (20.4)	1757 (20.1)
Level 4 (5000–9999)	2129 (34.0)	2105 (33.6)	10,686 (29.8)	10,659 (29.7)	2638 (30.2)	2531 (28.9)
Level 5 (≥10,000)	1445 (23.1)	1412 (22.6)	4390 (12.2)	4180 (11.7)	1339 (15.3)	1307 (14.9)
Hospitalisation (2011)	1217 (19.4)	1132 (18.1)	6512 (18.1)	5537 (15.4)	1376 (15.7)	1373 (15.7)
No. of hospitalised days (2011)						
Mean days (sd)	3.2 (12.4)	3.1 (12.0)	2.6 (10.4)	2.2 (8.9)	2.3 (10.3)	2.0 (8.4)
0–3 days	5284 (84.4)	5320 (84.9)	30,896 (86.1)	31,587 (88.0)	7715 (88.2)	7725 (88.3)
4–10 days	494 (7.9)	475 (7.6)	2678 (7.5)	2148 (6.0)	583 (6.7)	566 (6.5)
>10 days	485 (7.7)	468 (7.5)	2315 (6.5)	2154 (6.0)	451 (5.1)	458 (5.2)

ICM, integrated care model; SCM, standard care model; sd, standard deviation.

Overall, 19% of persons enrolled in integrated care models and 21% of controls (with standard care model) had at least one diabetes-related hospitalisation (total) in the following year among the patient group with diabetes (Table 3).

**Table 3:** Disease-related hospitalisation frequencies by chronic disease and care model (integrated vs standard) after propensity matching.

Hospitalisation	Diabetes		Cardiovascular diseases		Respiratory illnesses	

	With ICM *n* = 6263	With SCM *n* = 6263	With ICM *n* = 35,889	With SCM *n* = 35,889	With ICM *n* = 8749	With SCM *n* = 8749
	*n* (%)	*n* (%)	*n* (%)	*n* (%)	*n* (%)	*n* (%)

Diabetes related cause of hospitalisation (total)	1194 (19.1)	1298 (20.7)				
Diabetes mellitus	1135 (18.1)	1229 (19.6)				
Renal failure	383 (6.1)	377 (6.0)				
Retinal disorder and cataract	12 (0.2)	24 (0.4)				
Ischaemic heart disease	366 (5.8)	382 (6.1)				
Cerebrovascular disease	74 (1.2)	83 (1.3)				
Atherosclerosis	126 (2.0)	147 (2.4)				
Diabetic peripheral angiopathy	39 (0.6)	52 (0.8)				
Diabetic monoeuropathy	1 (0.02)	1 (0.02)				
Diabetic polyneuropathy	60 (1.0)	67 (1.1)				
Cardiovascular disease-related cause of hospitalisation (total)			5273 (14.7)	5508 (15.4)		
Hypertensive disease			4428 (12.3)	4673 (13.0)		
Ischaemic heart disease			1537 (4.3)	1566 (4.4)		
Pulmonary heart disease and diseases of pulmonary circulation			245 (0.7)	273 (0.8)		
Other form of heart diseases			1655 (4.6)	1706 (4.8)		
Cerebrovascular disease			339 (0.9)	367 (1.0)		
Atherosclerosis, aortic aneurysm and dissection and other aneurysm and dissection			509 (1.4)	589 (1.6)		
Peripheral vascular disease, unspecified			54 (0.2)	46 (0.1)		
Arterial embolism and thrombosis			54 (0.2)	72 (0.2)		
Respiratory illness-related cause of hospitalisation (total)					602 (6.9)	594 (6.8)
Simple and mucopurulent chronic bronchitis					4 (0.1)	3 (0.03)
Unspecified chronic bronchitis					12 (0.1)	9 (0.1)
Emphysema					9 (0.1)	6 (0.1)
Other chronic obstructive pulmonary disease					419 (4.8)	404 (4.6)
Asthma					174 (2.0)	194 (2.2)
Status asthmaticus					2 (0.02)	5 (0.1)
Bronchiectasis					14 (0.2)	7 (0.1)

ICM, integrated care model; SCM, standard care model.

In the diabetes cohort, renal failure (6.1 and 6.0% of all patients, respectively) and ischaemic heart disease (5.8 and 6.1% of all patients, respectively) were the most prevalent diabetes-related complications. Hypertensive disease was the most frequent cause of cardiovascular disease-related hospitalisation in the cohort with cardiovascular diseases (12 and 13% of all patients, respectively). Approximately 7% of the patients with respiratory illnesses were hospitalised caused by a respiratory-related illness in the follow-up year.

Table 4 shows the mean total healthcare costs for patients with and without integrated care models among all three patient groups. The cost difference between patients enrolled in integrated care models and those with standard care models amounted to CHF 1064 per patient/year in the diabetes cohort, CHF 680 in the cohort with cardiovascular diseases and CHF 501 in the respiratory illnesses cohort.

**Table 4:** Total healthcare costs by chronic disease and care model (integrated vs standard) after propensity matching.

Hospitalisation	Diabetes			Cardiovascular diseases			Respiratory illnesses		

	Total	With ICM *n* = 6263	With SCM *n* = 6263	Total	With ICM *n* = 35,889	With SCM *n* = 35,889	Total	With ICM *n* = 8749	With SCM *n* = 8749
	CHF	CHF	CHF	CHF	CHF	CHF	CHF	CHF	CHF

Total healthcare costs (mean, standard deviation)	10,000 (14379.7)	9466 (13418.6)	10,530 (15263.0)	7842 (13063.5)	7502 (12186.2)	8182 (13877.4)	7679 (14664.5)	7428 (13164.8)	7929 (16021.2)

ICM, integrated care model; SCM, standard care model.

Table 5 presents the results of the multivariate logistic regression models estimating the effect of integrated care (at baseline: 2012) on disease-related hospitalisations (at follow-up: 2013) for each cohort. After adjusting for sociodemographics, deductibles, comorbidity and previous hospitalisation, a significant effect of integrated care on disease-related hospitalisation could be observed among patients with diabetes (Odds Ratio: 0.87; 95% confidence interval: 0.79–0.95). For the cardiovascular diseases cohort, patients enrolled with an integrated care model had a lower risk of being hospitalised caused by a cardiovascular diseases than patients with a standard care model (Odds Ratio: 0.92, 95% confidence interval: 0.88–0.96). There was no significant difference in the probability of hospitalisation between patients with an integrated care model and patients with a standard care model among patients with respiratory illnesses. In all three patient groups, men, older patients and patients with higher chronic disease scores and higher number of hospitalised days in the previous year were significantly more likely to have a disease-related hospitalisation in the following year. There also seemed to be vast regional variations in the probability to be hospitalised.

**Table 5:** Prediction of disease-related hospitalisation by chronic disease on the propensity-matched cohort.

Characteristic	Hospitalisation (2013)					

	Diabetes		Cardiovascular diseases		Respiratory illnesses	

	Odds ratio	95% confidence interval	Odds ratio	95% confidence interval	Odds ratio	95% confidence interval

Population characteristics
Gender
Male	1.00		1.00		1.00	
Female	0.79***	0.72–0.87	0.75***	0.72–0.78	0.64***	0.57–0.73
Age group (years)
18–44	1.00		1.00		1.00	
45–54	1.49*	1.06–2.10	2.26***	1.84–2.78	1.99***	1.37–2.89
55–64	1.90***	1.39–2.60	3.42***	2.83–4.14	4.31***	3.12–5.96
65–74	2.47***	1.82–3.36	5.11***	4.24–6.16	6.54***	4.80–8.92
75–84	3.57***	2.62–4.85	7.25***	6.01–8.73	7.15***	5.21–9.82
≥85	5.11***	3.64–7.16	10.45***	8.62–12.65	9.19***	6.41–13.17
Region of residence
Lake Geneva	1.00		1.00		1.00	
Mittelland	1.91***	1.49–2.44	2.11***	1.84–2.43	1.75***	1.27–2.40
Northwest	2.22***	1.74–2.83	2.53***	2.21–2.91	2.12***	1.55–2.90
East	2.21***	1.73–2.83	2.41***	2.09–2.77	2.02***	1.46–2.79
Ticino	6.62**	1.84–23.78	1.82	0.89–3.71	2.85	0.77–10.60
Central	1.90***	1.37–2.64	2.20***	1.85–2.62	1.67*	1.07–2.62
Zurich	1.93***	1.50–2.46	2.34***	2.04–2.69	2.10***	1.54–2.87
Health insurance status
Care model
Standard care model	1.00		1.00		1.00	
Integrated care model	0.87**	0.79–0.95	0.92***	0.88–0.96	0.95	0.84–1.07
Deductible
Low (CHF 300/500)	1.00		1.00		1.00	
High (>CHF500)	0.89	0.70–1.13	0.79***	0.73–0.86	0.56***	0.42–0.75
Chronic Disease Score
Level 1 (0–999)	1.00		1.00		1.00	
Level 2 (1000–2499)	1.33	0.96–1.84	1.25***	1.14–1.36	1.15	0.81–1.63
Level 3 (2500–4999)	1.66**	1.20–2.32	1.49***	1.37–1.61	1.51**	1.11–2.05
Level 4 (5000–9999)	2.33***	1.69–3.20	2.02***	1.87–2.18	1.93***	1.44–2.58
Level 5 (≥10,000)	3.51***	2.55–4.84	2.92***	2.68–3.18	3.05***	2.264–4.12
No. of hospitalised days (2011)
0–3 days	1.00		1.00		1.00	
4–10 days	1.28**	1.10–1.50	1.47***	1.37–1.59	1.77***	1.46–2.15
>10 days	1.92***	1.66–2.23	1.88***	1.75–2.02	2.66***	2.22–3.19

*p < 0.05; **p < 0.01; ***p < 0.01.

Table 6 shows the adjusted mean cost differences between enrolees in integrated care models and those enrolled in standard care models for each patient cohort, controlled for sociodemographics and morbidity indicators. The total healthcare costs were almost CHF 780 lower in patients with integrated care models (coefficient: –777.8, 95% confidence interval: –1040.8 to –516.5) compared with patients without integrated care models in the diabetes cohort.

**Table 6:** Prediction of total healthcare costs by chronic disease on the propensity-matched cohort.

Characteristic	Healthcare costs (2013)					

	Diabetes		Cardiovascular diseases		Respiratory illnesses	

	Estimate CHF	95% confidence interval	Estimate CHF	95% confidence interval	Estimate CHF	95% confidence interval

Population characteristics
Gender
Male	Ref.		Ref.		Ref.	
Female	–669.1***	–933.3 to –403.4	–412.2***	–499.0 to –325.8	284.0***	166.3–402.3
Age group (years)
18–44	Ref.		Ref.		Ref.	
45–54	–769.7**	–1308.6 to –248.5	213.4**	59.9–364.6	220.4**	65.7–386.0
55–64	–84.5	–632.4–438.5	431.2***	284.9–573.9	1127.3***	896.3–1373.2
65–74	740.1**	190.0–1264.2	1242.4***	1090.0–1391.7	1942.7***	1642.0–2259.3
75–84	1850.8***	1252.8–2430.5	2300.5***	2126.5–2473.1	2655.6***	2230.1–3108.7
≥85	3379.8***	2447.3–4370.3	3499.8***	3226.6–3780.0	3368.2***	4338.1–2517.0
Region of residence
Lake Geneva	Ref.		Ref.		Ref.	
Mittelland	–555.5	–1138.5 to –10.6	–221.6*	–436.4 to –17.0	–9.9	–209.0 to 180.6
Northwest	188.5	–396.6 to 735.4	–262.4*	–476.4 to –58.5	225.3*	11.7–435.9
East	61.2	–535.0 to 622.1	–522.2***	–737.1 to –317.2	–251.9*	–454.7 to –53.1
Ticino	6705.3	–1399.7 to 25930.6	–1006.0	–2111.3 to 962.9	–944.6***	–506.6 to –1227.3
Central	81.5	–740.1 to 948.0	–283.4*	–548.6 to –17.4	–68.5	–326.1 to 217.4
Zurich	–96.4	–687.6 to 457.9	12.7	–202.7 to 218.2	145.4	–56.3 to 340.4
Health insurance status
Care model
Standard care model	Ref.		Ref.		Ref.	
Integrated care model	–777.8***	–1040.8 to –516.5	–441.3***	–527.3 to –355.6	–217.9***	–340.2 to –96.6
Deductible
Low (CHF 300/500)	Ref.		Ref.		Ref.	
High (>CHF500)	–344.2	–819.8 to 180.2	–1051.0***	–1150.7 to –950.0	–1266.0***	–1407.7 to –1127.0
Chronic disease score
Level 1 (0–999)	Ref.		Ref.		Ref.	
Level 2 (1000–2499)	642.2**	205.0–1060.8	861.4***	749.1–974.9	1054.1***	878.1–1238.6
Level 3 (2500–4999)	2189.6***	1698.1–2674.4	2019.8***	1894.5–2146.2	1855.1***	1628.2–2090.9
Level 4 (5000–9999)	3750.8***	3271.0–4216.0	4405.9***	4251.7–4561.7	4820.9***	4489.9–5161.5
Level 5 (≥10,000)	10022.8***	9322.7–10734.6	11260.3***	10877.5–11654.4	11960.6***	11197.6–12766.9
No. of hospitalised days (2011)
0–3 days	Ref.		Ref.		Ref.	
4–10 days	2809.3***	2106.0–3575.4	1768.7***	1506.2–2044.2	1644.4***	1137.8–2223.9
>10 days	6260.6***	5206.3–7407.3	5030.8***	4592.6–5489.8	7079.3***	5810.9–8492.1

Ref., reference. *p < 0.05; **p < 0.01; ***p < 0.01.

The adjusted mean healthcare costs were also estimated for patients with cardiovascular diseases and those with respiratory illnesses. For the cardiovascular diseases cohort, the cost difference between patients with and without integrated care models amounted to about CHF 440 (coefficient: –441.3, 95% confidence interval: –527.3 to –355.6), and for the respiratory illnesses cohort, approximately CHF 220 (coefficient: –217.9, 95% confidence interval: –340.2 to –96.6). The cost difference increased substantially with age, severity of comorbidity and the number of hospitalised days in the previous year in all three patient groups.

## Discussion

In this propensity-matched cohort analysis of a large health insurance database in Switzerland we found a substantial effect of integrated care on medical expenditures and disease-related hospitalisations reflecting quality of care in patients suffering from highly prevalent chronic diseases.

The first major finding of our study was that the enrolment in an integrated care model predicts a significantly decreased probability of future disease-related hospitalisation, reflecting quality of care, among patients with diabetes and cardiovascular diseases, compared with patients enrolled in a standard care model. A difference in hospitalisation risk among patients with respiratory illnesses could not be proved. These results are in line with a recently published meta-review from Martínez-González et al. [4]. Despite remarkable heterogeneity in the methodological quality of the included reviews, the authors found positive effects of integrated care programmes regarding hospital admissions and readmissions among patients with chronic heart failure and diabetes, and only little evidence on beneficial effects regarding guideline adherence for patients with chronic obstructive pulmonary disease. We assume that some elements of the concept of ‘integrated care’ (e.g. guideline-based treatment) have already been stronger implemented in the care of diabetes and cardiovascular diseases than in chronic obstructive pulmonary disease and asthma in the Swiss general practice. Furthermore, our results underline findings from interventions studies showing that a multicomponent guide for chronic illness leads to better intermediate outcomes in chronically ill patients. For example, a systematic US review suggested that healthcare organisations, which have used a multicomponent template for chronic illness management (the so-called ‘Chronic Care Model’), improve the quality of patient care and lead to better health outcomes in chronically ill patients [23]. Consistent with US findings, a recent national study could show an improved diabetes management from patients’ perspective in Chronic Care Model-orientated managed care organisations compared to usual primary care [24]. However, only a few comparable national studies examining the effect of integrated care on outcomes such as disease-related hospitalisations, reflecting quality of care, are available. One study suggested that patients in managed care health plans had a higher rate of potentially inappropriate medication use, considered as quality proxy, and also a higher risk to be hospitalised [10]. A federal report could show that outpatient hospital visits caused by trivial disorders were lower in patients enrolled in contracted integrated care models with capitation [25].

The second finding of our study was that integrated care reduces healthcare costs up to 10% in all three patient groups with chronic diseases, but with highest impact in patients with diabetes. Although the international evidence for cost-effectiveness is weak [4], some national studies could find an association between integrated care models and reduced costs. So our finding are in line with previous studies conducted in the Swiss healthcare system, which reported a cost reduction between about 10 and 20% [5,6]. However, these studies investigated the cost-effectiveness for the whole population and did not differentiate between the most prevalent chronic diseases. Since chronic diseases, such as diabetes, cardiovascular diseases and respiratory illnesses, are frequently occurring and cost-intensive for the healthcare system, integrated care models may provide an effective strategy to improve the quality of care and to reduce healthcare costs in chronically ill patients. Our study findings contribute to the ongoing political discussion on integrated care and provide evidence for an improved and more effective care of patients with chronic diseases. In this context it is also important to stress the fact that integrated care models in our study were defined as a general practitioner-centred model or Health Maintenance Organisation model, in which the coordinating provider compulsorily coordinates the medical care during the treatment process. In view of the fact that the implementation of guidelines is not standard in Swiss general practice, it is assumed that the corresponding physicians used in varying degrees in evidence-based guidelines in the treatment of chronic diseases. Thus, our results suggest that this organisational structure in healthcare management is already able to decrease the hospitalisation risk as well as costs. A nationwide implementation of guidelines such as elements used in the Chronic Care Model would improve the patient care even more, in terms of reduction of the disease severity or of avoiding the occurrence of comorbidity, and, furthermore, increase the cost-effectiveness.

Several strengths and limitations of our study have to be taken into account. The main strength is that the study is based on a comprehensive healthcare claims database, which covers a large population including patients with and without enrolment in integrated care models. Furthermore, this study is, to the best of our knowledge, the first one that investigated the impact of integrated care on quality indicators, in terms of disease-related hospitalisation, in patients with chronic diseases. The study also has several limitations. First, the number of patients with diabetes, cardiovascular diseases and respiratory illnesses may be each biased because clinical diagnoses (e.g., International Classification of Diseases, Version 10) from the outpatient setting were not available. However, diagnoses based on prescribed drugs are a valid proxy for clinical diagnoses and widely used in epidemiological and outcomes research to assess prevalence [18,22]. For example, Cossman et al. [26] showed that prescription data are a useful proxy for disease-specific prevalence. Also, Chini et al. [27] concluded that drug data are a reliable source for prevalence estimates of chronic conditions. Especially the use of prescriptions for anti-diabetic drugs and for chronic obstructive pulmonary disease drugs respectively could be used for a precise identification of patients with diabetes [28,29] and patients with chronic obstructive pulmonary disease respectively [30] across large populations. Second, although using drug data as proxy diagnosis is a valid approach, it did not allow us to differentiate between particular diseases within each chronic disease group. Some elements (WHO Anatomical Therapeutic Chemical codes) could not be uniquely assigned to a given disease. Within the diabetes group, it was not possible to distinguish between diabetes type 1 and type 2, within the group with cardiovascular diseases, between e.g. hypertension and other heart diseases, and within the respiratory illnesses group, between asthma and chronic obstructive pulmonary disease. Therefore, we were limited in the selection of chronic diseases and not able to consider the same categories of chronic patients as used in the study of Martínez-González et al. [4]. On the other side, we were able to identify three highly prevalent chronic diseases, where especially integrated care might makes sense and can help to avoid worsening health or to prevent the development of (further) comorbidities. Third, we are aware of the fact of potentially mismeasured or missing covariates such as variables indicating patients’ health status. Since medical diagnoses from the outpatient setting and further clinical parameters (e.g. laboratory values, body mass index, smoking) are not available in our data, we used drug-based diagnoses as a proxy for clinical diagnoses. For example, to measure patients’ comorbidity we used the Chronic Disease Score, a prescription-based morbidity measure. The included comorbidities may be biased because not all WHO Anatomical Therapeutic Chemical codes could be uniquely assigned to the treatment of a given disease. However, the Chronic Disease Score is the most frequently used and validated prescription-based method to determine chronic morbidity. Fourth, we did not distinguish between patients suffering from two or three chronic diseases simultaneously (diabetes, cardiovascular diseases, respiratory illnesses); thus, it is possible that for example a patient with diabetes belongs to the group with diabetes as well as to the group with cardiovascular diseases. However, we are quite confident that the allocation has not greatly influenced our results, since we adjusted for the comorbidity status (including diabetes, cardio vascular disease, asthma/chronic obstructive pulmonary disease, respectively) in each of the analysis. Fifth, estimates of healthcare cost may be slightly too low because approximately 1.5% of the healthcare costs were not paid by the health insurer but directly by the patient (out-of-pocket).

## Conclusion

These study findings contribute to the ongoing political discussion on the efficiency and also the further promotion of integrated care models and provide valuable information for an improved and more effective care of patients with chronic diseases. Integrated care should be seen as a key issue in healthcare quality and also more addressed in clinical, public health and health policy debates.

## Competing Interests

The authors declare that they have no competing interests.

## Appendix

**Table A1:** Classification of patients with chronic diseases

Chronic disease	Identification via ATC code	Identification via ICD-10 code

Diabetes mellitus	A10A, A10B, A10X	E10–14
Cardiovascular diseases	B01AA, B01AC, B01AX, B01AE07, C01–03, C04A, C07–09	I10–I15, I20–I25, I26–I28, I47–I50, I61–I66, I67.0, I67.2, I67.4, I70–72, I73.9, I74
Respiratory illnesses	R03	J41–46

**Table A2:** Classification of disease-related hospitalisations.

Chronic disease	Disease-related hospitalisation according ICD-10 classification

Diabetes mellitus	E10–E14, I20–I25, I61–I66, I67.0, I67.2, I67.4, N17–N19, N08.3, H28.0, H36.0, I70, I79.2, G59.0, G63.2
Cardiovascular diseases	I10–I15, I20–I28, I47–I50, I61–I66, I67.0, I67.2, I67.4, I70–I72, I73.9, I74
Respiratory illnesses	J41–J47 (excl. J43.0)

**Table A3:** Patients characteristics by chronic disease before propensity-matching.

Characteristic	Patients with diabetes *n* = 36,532	Patients with cardiovascular diseases *n* = 186,986	Patients with respiratory illnesses *n* = 45,364
	*n* (%)	*n* (%)	*n* (%)

Population characteristics (2012)
Male	20052 (54.9)	85832 (45.9)	19447 (42.9)
Mean age (sd)	67.2 (13.0)	68.2 (13.7)	58.9 (18.1)
Age group (years)
18–44	2033 (5.6)	10455 (5.6)	10447 (23.0)
45–54	3739 (10.2)	18727 (10.0)	6633 (14.6)
55–64	7929 (21.7)	37159 (19.9)	8302 (18.3)
65–74	11059 (30.3)	52590 (28.1)	9970 (22.0)
75–84	9376 (25.7)	49459 (26.5)	7714 (17.0)
≥85	2396 (6.6)	18596 (10.0)	2298 (5.1)
Region of residence
Lake Geneva	5465 (15.0)	23980 (12.8)	7203 (15.9)
Mittelland	7208 (19.7)	35900 (19.2)	8601 (19.0)
Northwest	5732 (15.7)	29611 (15.8)	7067 (15.6)
East	4966 (13.6)	26843 (14.4)	5695 (12.6)
Ticino	2539 (7.0)	12355 (6.6)	2879 (6.4)
Central	2476 (6.8)	1337 (7.1)	3051 (6.7)
Zurich	8146 (22.3)	44960 (24.0)	10868 (24.0)
Health insurance status
Integrated care model	6263 (17.1)	35889 (19.2)	8749 (19.3)
Deductible
High (>CHF500)	1775 (4.9)	17125 (9.2)	4951 (10.9)
Low (CHF300/500)	34757 (94.1)	169861 (90.8)	40413 (89.1)
Chronic disease score (CDS)
Mean CDS (sd)	7746 (6128.9)	5713 (5250.8)	6823 (6113.6)
Level 1 (0–999)	1616 (4.4)	25742 (13.8)	6874 (15.2)
Level 2 (1000–2499)	6880 (18.8)	30316 (16.2)	5922 (13.1)
Level 3 (2500–4999)	5671 (15.5)	42423 (22.7)	8388 (18.5)
Level 4 (5000–9999)	12394 (33.9)	59855 (32.0)	14736 (32.5)
Level 5 (≥10000)	9971 (27.3)	28650 (15.3)	9444 (20.8)
Hospitalisation (2011)	7564 (20.7)	36747 (19.7)	8952 (19.7)
No. of hospitalised days (2011)
Mean days (sd)	3.7 (13.8)	3.3 (12.8)	3.4 (13.3)
0–3 days	30356 (83.1)	157966 (84.5)	38311 (84.5)
4–10 days	2827 (7.7)	14028 (7.5)	3387 (7.5)
>10 days	3349 (9.2)	14992 (8.0)	3666 (8.1)

**Table A4:** Results showing the effectiveness of the propensity score matching among patients with diabetes.

	**Summary of balance for all data**						

	**Means Treated**	**Means Control**	**SD Control**	**Mean Diff**	**eQQ Med**	**eQQ Mean**	**eQQ Max**

Distance	0.2134	0.1627	0.0854	0.0507	0.0583	0.0507	0.0942
Age in years	67.1062	67.2526	13.0728	–0.1464	1	0.6077	3
CDS	7140.5362	7871.5302	6175.6716	–730.9941	775	741.2724	4399
Ded_high	0.0497	0.0484	0.2145	0.0013	0	0.0013	1
Geneva	0.065	0.1671	0.3731	–0.1021	0	0.1022	1
Mittelland	0.2071	0.1953	0.3964	0.0118	0	0.0118	1
Northwest	0.2612	0.1353	0.3421	0.1259	0	0.1258	1
East	0.2087	0.1209	0.326	0.0878	0	0.0878	1
Ticino	0.0008	0.0837	0.277	–0.0829	0	0.083	1
Central	0.0375	0.074	0.2618	–0.0365	0	0.0366	1
Zurich	0.2197	0.2237	0.4167	–0.004	0	0.004	1
No. of hospitalised days	3.243	3.8468	14.0993	–0.6038	0	0.6302	189
	**Summary of balance for matched data**						

	**Means Treated**	**Means Control**	**SD Control**	**Mean Diff**	**eQQ Med**	**eQQ Mean**	**eQQ Max**

Distance	0.2134	0.2134	0.068	0	0	0	4.00E-04
Age in years	67.1062	67.0564	12.551	0.0498	0	0.5307	4.00E+00
CDS	7140.5362	6966.2443	5491.9203	174.2919	27	178.753	1.67E+04
Ded_high	0.0497	0.0474	0.2126	0.0022	0	0.0022	1.00E+00
Geneva	0.065	0.0664	0.249	–0.0014	0	0.0014	1.00E+00
Mittelland	0.2071	0.2058	0.4043	0.0013	0	0.0013	1.00E+00
Northwest	0.2612	0.2561	0.4365	0.0051	0	0.0051	1.00E+00
East	0.2087	0.2109	0.408	–0.0022	0	0.0022	1.00E+00
Ticino	0.0008	0.0008	0.0282	0	0	0	0.00E+00
Central	0.0375	0.0367	0.1881	0.0008	0	0.0008	1.00E+00
Zurich	0.2197	0.2232	0.4164	–0.0035	0	0.0035	1.00E+00
No. of hospitalised days	3.243	3.0838	12.0279	0.1592	0	0.179	2.50E+01
	**Percent Balance Improvement**						

	**Mean Diff.**	**eQQ Med**	**eQQ Mean**	**eQQ Max**			

Distance	99.9961	99.998	99.9881	99.5987			
Age in years	65.9777	100	12.6642	–33.3333			
CDS	76.1569	96.5161	75.8857	–280.3137			
Ded_high	–73.2294	0	–75	0			
Geneva	98.5928	0	98.5938	0			
Mittelland	89.1814	0	89.1892	0			
Northwest	95.9416	0	95.9391	0			
East	97.4541	0	97.4545	0			
Ticino	100	0	100	100			
Central	97.8136	0	97.8166	0			
Zurich	11.2542	0	12	0			
No. of hospitalised days	73.6366	0	71.5987	86.7725			

**Table A5:** Results showing the effectiveness of the propensity score matching among patients with cardiovascular diseases.

	**Summary of balance for all data**						

	**Means Treated**	**Means Control**	**SD Control**	**Mean Diff**	**eQQ Med**	**eQQ Mean**	**eQQ Max**

Distance	0.2337	0.182	0.0916	0.0517	0.0463	0.0517	0.1126
Age in years	67.9767	68.2904	13.7438	–0.3137	1	0.5933	3
CDS	5121.7543	5853.2708	5314.8696	–731.5165	723	731.95	8342
Ded_high	0.1071	0.0879	0.2831	0.0192	0	0.0192	1
Geneva	0.0506	0.1467	0.3538	–0.0961	0	0.0961	1
Mittelland	0.2045	0.189	0.3915	0.0155	0	0.0155	1
Northwest	0.2474	0.1372	0.3441	0.1102	0	0.1102	1
East	0.2091	0.128	0.3341	0.0812	0	0.0812	1
Ticino	0.0009	0.0816	0.2737	–0.0806	0	0.0806	1
Central	0.0382	0.0792	0.2701	–0.041	0	0.041	1
Zurich	0.2492	0.2384	0.4261	0.0109	0	0.0109	1
No. of hospitalised days	2.5988	3.4439	13.2891	–0.8451	0	0.8484	126
	**Summary of balance for matched data**						

	**Means Treated**	**Means Control**	**SD Control**	**Mean Diff**	**eQQ Med**	**eQQ Mean**	**eQQ Max**

Distance	0.2337	0.2337	0.0662	0	0	0	0.0003
Age in years	67.9767	67.8084	13.5411	0.1683	0	0.4208	2
CDS	5121.7543	4974.4921	4685.194	147.2622	37	147.2622	9616
Ded_high	0.1071	0.104	0.3052	0.0031	0	0.0031	1
Geneva	0.0506	0.0516	0.2212	–0.001	0	0.001	1
Mittelland	0.2045	0.2063	0.4047	–0.0018	0	0.0018	1
Northwest	0.2474	0.2422	0.4284	0.0053	0	0.0053	1
East	0.2091	0.2083	0.4061	0.0008	0	0.0008	1
Ticino	0.0009	0.0009	0.0303	0	0	0	0
Central	0.0382	0.0378	0.1907	0.0004	0	0.0004	1
Zurich	0.2492	0.2529	0.4347	–0.0037	0	0.0037	1
No. of hospitalised days	2.5988	2.2298	8.943	0.369	0	0.369	92
	**Percent Balance Improvement**						

	**Mean Diff.**	**eQQ Med**	**eQQ Mean**	**eQQ Max**			

Distance	99.999	100	99.9974	99.7426			
Age in years	46.348	100	29.0753	33.3333			
CDS	79.8689	94.8824	79.8808	–15.2721			
Ded_high	83.6103	0	83.5994	0			
Geneva	98.9564	0	98.9565	0			
Mittelland	88.3166	0	88.3094	0			
Northwest	95.2223	0	95.2224	0			
East	98.9701	0	98.9701	0			
Ticino	100	0	100	100			
Central	99.1171	0	99.1174	0			
Zurich	66.4629	0	66.4962	0			
No. of hospitalised days	56.3321	0	56.5015	26.9841			

**Table A6:** Results showing the effectiveness of the propensity score matching among patients with respiratory illnesses.

	**Summary of balance for all data**						

	**Means Treated**	**Means Control**	**SD Control**	**Mean Diff**	**eQQ Med**	**eQQ Mean**	**eQQ Max**

Distance	0.225	0.1852	0.0811	0.0398	0.0311	0.0398	0.1281
Age in years	57.0702	59.2733	17.9302	–2.2031	1	2.2023	6
CDS	5843.5821	7056.6819	6215.7646	–1213.0998	1274	1214.3879	5390
Ded_high	0.1458	0.1004	0.3005	0.0455	0	0.0455	1
Geneva	0.1088	0.1707	0.3763	–0.0619	0	0.0619	1
Mittelland	0.2145	0.1836	0.3872	0.0309	0	0.0309	1
Northwest	0.2277	0.1386	0.3455	0.0891	0	0.089	1
East	0.1648	0.1162	0.3204	0.0487	0	0.0487	1
Ticino	0.0015	0.0783	0.2686	–0.0768	0	0.0768	1
Central	0.0405	0.0737	0.2612	–0.0332	0	0.0333	1
Zurich	0.2422	0.2389	0.4264	0.0033	0	0.0032	1
No. of hospitalised days	2.2707	3.6888	13.9077	–1.4181	0	1.427	86
	**Summary of balance for matched data**						

	**Means Treated**	**Means Control**	**SD Control**	**Mean Diff**	**eQQ Med**	**eQQ Mean**	**eQQ Max**

Distance	0.225	0.225	0.0616	0	0	0	1.80E-03
Age in years	57.0702	56.3366	18.3466	0.7336	1	0.9846	4.00E+00
CDS	5843.5821	5560.6678	5161.4738	282.9143	118	282.9143	1.46E+04
Ded_high	0.1458	0.1423	0.3494	0.0035	0	0.0035	1.00E+00
Geneva	0.1088	0.1182	0.3228	–0.0094	0	0.0094	1.00E+00
Mittelland	0.2145	0.2161	0.4116	–0.0016	0	0.0016	1.00E+00
Northwest	0.2277	0.2203	0.4144	0.0074	0	0.0074	1.00E+00
East	0.1648	0.1561	0.363	0.0087	0	0.0087	1.00E+00
Ticino	0.0015	0.0015	0.0385	0	0	0	0.00E+00
Central	0.0405	0.0385	0.1925	0.0019	0	0.0019	1.00E+00
Zurich	0.2422	0.2493	0.4326	–0.0071	0	0.0071	1.00E+00
No. of hospitalised days	2.2707	2.0296	8.3597	0.2411	0	0.2525	6.50E+01
	**Percent Balance Improvement**						

	**Mean Diff.**	**eQQ Med**	**eQQ Mean**	**eQQ Max**			

Distance	99.9864	99.9948	99.9755	98.6262			
Age in years	66.7028	0	55.2938	33.3333			
CDS	76.6784	90.7378	76.7031	–171.4286			
Ded_high	92.2086	0	92.2111	0			
Geneva	84.8611	0	84.8708	0			
Mittelland	94.8211	0	94.8148	0			
Northwest	91.6597	0	91.656	0			
East	82.1497	0	82.1596	0			
Ticino	100	0	100	100			
Central	94.1467	0	94.1581	0			
Zurich	–117.8243	0	–121.4286	0			
No. of hospitalised days	83.0015	0	82.3068	24.4186			
